# Analysis of the Mechanism of GuizhiFuling Wan in Treating Adenomyosis Based on Network Pharmacology Combined with Molecular Docking and Experimental Verification

**DOI:** 10.1155/2022/6350257

**Published:** 2022-08-26

**Authors:** Yaxin Shi, Chengyuan Zhang, Xin Wang, Zilu Wang, Yiran Zhang, Zhiyong Liu, Xin Wang, Wei Shi

**Affiliations:** ^1^College of Traditional Chinese Medicine, Shandong University of Traditional Chinese Medicine, Jinan 250355, China; ^2^Guangzhou University of Chinese Medicine, Guangzhou 510006, China; ^3^Affiliated Hospital of Shandong University of Traditional Chinese Medicine, Jinan 250011, China

## Abstract

**Background:**

The effect of GuizhiFuling Wan (GFW) on adenomyosis (AM) is definite. This study aimed to explore the mechanism and key therapeutic targets of GFW in treating AM through network pharmacology combined with molecular docking and experimental verification.

**Materials and Methods:**

In network pharmacology, firstly, the active components of GFW, its drug, and disease targets were screened through several related public databases, and GFW-AM common targets were obtained after the intersection. Then, the biological function (Gene Ontology, GO) and pathway (Kyoto Encyclopedia of Genes and Genomes, KEGG) of GFW in treating AM were enriched and analyzed. Finally, the interaction and binding force between key components and key targets of GFW were verified by molecular docking. In the animal part, the effect of GFW on the expression of matrix metallopeptidase 2 (MMP-2), matrix metallopeptidase 9 (MMP-9), and vascular endothelial growth factor (VEGF) in mice with AM was observed by HE staining, ELISA, and immunohistochemistry.

**Results:**

In this study, 89 active components of GFW, 102 related targets, and 291 targets of AM were collected. After the intersection, 26 common targets were finally obtained. The key active compounds were baicalein, sitosterol, and *β*-sitosterol, and the key targets were MMP-2, MMP-9, and VEGF. GO and KEGG enrichment analyses showed that biological processes such as the positive regulation of vascular endothelial migration and signaling pathways such as TNF and HIF-1 were involved in regulating angiogenesis, invasion, and metastasis in AM. The molecular docking results showed that baicalein, *β*-sitosterol, and stigmasterol had better binding potential with MMP-2, MMP-9, and VEGF. The results of in vivo analysis showed that GFW could decrease the serum content and protein expression of MMP-2, MMP-9, and VEGF in mice with AM.

**Conclusions:**

GFW could reduce the expression of MMP-2, MMP-9, and VEGF, which might be an essential mechanism for GFW to inhibit the invasion and metastasis of ectopic tissues of AM.

## 1. Introduction

Adenomyosis (AM) refers to a disease in which active endometrial tissue (glands and stroma) appears in the myometrium of the uterus [1]. It is often manifested as abnormal uterine bleeding, dysmenorrhea, subfertility, and other symptoms, seriously affecting the life of patients [2, 3]. Nevertheless, many aspects of AM pathogenesis remain poorly characterized. It is generally believed to be closely related to the invasion and metastasis of the eutopic endometrium to the ectopic lesions caused by the invagination of the endometrial basal layer and the repair of tissue damage [4, 5]. The incidence of AM has gradually increased in recent years, and there is still a lack of effective treatment. The most common treatments in Western medicine are drug therapy and surgical treatment. Drug therapy mainly includes gonadotropin-releasing hormone analogs and oral contraceptives. Although the drugs mentioned above can relieve the symptoms of AM, they are accompanied by a series of side reactions such as irregular vaginal bleeding and a high recurrence rate [6]. Hysterectomy is the primary method for diagnosing and curing AM, but it is difficult for women of reproductive age to undergo hysterectomy [7, 8]. In recent years, the application of traditional Chinese medicine (TCM) syndrome differentiation therapy and the advantages of multicomponents, multi-targets, negligible side effects, etc., have achieved remarkable results in treating AM, which is an indispensable treatment means [9]. The Chinese expert consensus on the diagnosis and treatment of AM in 2020 clearly stated that TCM could be used to relieve pain caused by AM [10].

**Figure 1:**
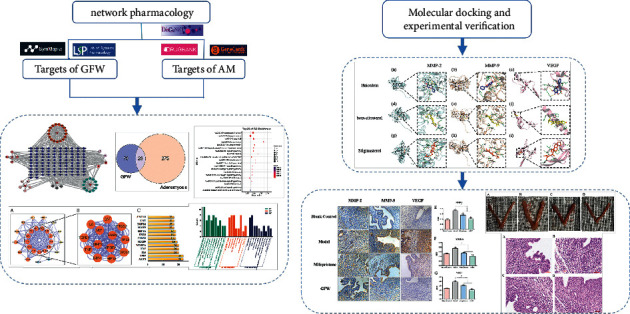
The flow chart of the study.

*GuizhiFuling Wan* (GFW), composed of *Cinnamon Twig*, *Poria*, *Red Peony*, *Peach Kernel*, and *Moutan Bark*, is a classic prescription created by Zhang Zhongjing in the Eastern Han Dynasty. With the function of dredging collaterals and relieving stasis, it is often used to treat dysmenorrhea, pelvic pain, menorrhagia, and other blood stasis diseases, and it has obvious therapeutic advantages for AM. Modern pharmacological studies showed that GFW could reduce the expression of vascular endothelial growth factor (VEGF), interleukin-2 (IL-2), and interleukin-8 (IL-8) in serum [11] and was found to be effective in rat models of AM [12, 13]. Wang verified that GFW played a role in treating AM by regulating the estrogen signaling pathway [14]. Our group also performed some research on GFW in treating AM. Through data mining, we found that *Red Peony*, *Poria*, and *Peach Kernel* were used more frequently in treating AM [15]. In vivo experiments in mice showed that GFW could increase the expression levels of caspase-3 and caspase-9, thereby promoting the apoptosis of ectopic intima [16]. Although GFW has become the focus of research, few studies have paid attention to the invasion and migration of AM. Moreover, GFW has the characteristics of multiple compounds and targets, which makes further research difficult. For the past few years, exploring the association between TCM prescriptions and complex diseases using the network pharmacology method to combine TCM prescriptions with molecular biological networks has become a research hotspot; it also provides new ideas for analyzing the compatibility of TCM compounds [17, 18]. However, some deficiencies have gradually emerged with the deepening of network pharmacology research, such as irregular data extraction, false positives in network prediction, and lack of follow-up experimental verification. Referring to the “Guide to Evaluation Methods of Network Pharmacology” [19], on the premise of reliable, standardized, and reasonable data collection and network analysis, this study used the network pharmacology method to predict the complex molecular mechanism of GFW in treating AM. In addition, the AM mouse model was established, which verified the drug substance basis and molecular mechanism of GFW intervention in AM, improved the reliability and rationality of the results, and provided the modern theoretical basis and new research ideas for further digging the target of GFW treatment in AM and subsequent clinical research. The specific process is shown in Figure 1.

## 2. Materials and Methods

### 2.1. Network Pharmacology

#### 2.1.1. Active Compounds and Targets of GFW

The chemical constituents of *Cinnamon Twig*, *Poria*, *Red Peony*, *Peach Kernel*, and *Cortex Moutan* in GFW were searched in the TCMSP database (https://tcmspw.com/tcmsp.php) [20] andSymMap database (https://www.symmap.org/) [21]. According to the oral bioavailability (OB) ≥ 30% and druglikeness (DL) ≥ 0.18 [20], the active components and targets of GFW were screened and added to refer to the published literature. Subsequently, the names of the targets were standardized using the UniProt database (https://www.uniprot.org/) [22].

#### 2.1.2. Collection of AM Targets

The keyword “adenomyosis” is entered into the GeneCards database (https://www.genecards.org) [23], the DisGeNET database (https://www.disgenet.org) [24], and the DrugBank database (https://www.drugbank.ca) [25] to obtain AM targets. After removing duplicate targets, AM targets were identified. Then, AM targets were converted to gene symbols using the UniProt database.

#### 2.1.3. Construction of a Network of GFW Active Compounds and a Network of Protein-Protein Interactions (PPI)

The active compounds and targets of GFW were uploaded into Cytoscape 3.7.2 (Institute of Systems Biolog, USA) [26] to generate network diagrams. The Venn diagram (https://www.bioinformatics.com.cn) was used to obtain the GFW-AM common targets. Then the common targets were uploaded to the STRING database (https://string-db.org/) [27]. The species selected were “human,” and the minimum confidence level was 0.4. The corresponding PPI network files were downloaded, and Cytoscape 3.7.2 was used to analyze the network. Also, the key targets were selected whose nodal degree value was higher than the median.

#### 2.1.4. Gene Ontology (GO) and Kyoto Encyclopedia of Genes and Genomes (KEGG) Enrichment Analysis

The GFW and AM key targets were imported into the DAVID database (https://david.ncifcrf.gov/) [28] to perform enrichment analysis of GO and KEGG pathways. The species was “human” with *P* < 0.05 as the standard. *P* values were sorted from small to large, and the results of the GO and KEGG enrichment analyses were selected by *P* value to determine the top 10 and top 20, respectively.

#### 2.1.5. Molecular Docking Verification

To verify the accuracy of network pharmacology, key components in GFW were selected for molecular docking with key targets of AM. The mol2 structures of the ligands were taken from TCMSP, and the 3D structures of the receptors were taken from UniProt (https://www.uniprot.org/). Ligands and receptors were optimized using ADFRsuite and converted to pdbqt structures. Then, the binding potential of ligands and receptors was evaluated using the molecular docking of Autodock Vina [29]. The smaller the binding energy, the more stable the binding of ligands and receptors. When the binding energy is less than −5.0 kcal/mol, the ligands and receptors are considered to have a better binding capacity [30].

### 2.2. Experimental Verification

#### 2.2.1. Animals

The experiment was approved by the Ethics Committee of Affiliated Hospital of Shandong University of Traditional Chinese Medicine (approval number AWE-2019-010). A total of 15 healthy SPF 6- to 7-week-old institute of cancer research (ICR) mice, including 10 female nulliparous mice and 5 male mice, were obtained from Beijing WeitongLihua Laboratory Animal Technology Co., Ltd. (License number: SCXK, Beijing 2016-0006). Raising environment: all mice were raised in the SPF experimental animal center of Shandong University of Traditional Chinese Medicine. They were housed in standard cages with a 12/12-hour light-dark cycle (lights on from 08:00 to 20:00) and had free access to food and water. Indoor temperature was controlled at 24 ± 2°C and relative humidity was 60%–65%.

#### 2.2.2. Experimental Drugs

Chinese patent medicine GFW (6 g/pill) was provided by Shanxi Tiansheng Pharmaceutical Co., Ltd. (batch number Z14020791); mifepristone (25 mg/tablet) was manufactured by Zhejiang Xianju Pharmaceutical Co., Ltd. (batch number 0950347); tamoxifen citrate (10 mg/tablet) was manufactured by Shandong Health Pharmaceutical Co., Ltd. (batch number H37022925); baicalein reference standards (HPLC ≥ 98%, batch number SS8010), *β*-sitosterol reference standards (HPLC ≥ 98%, batch number SS8580), stigmasterol reference standards (HPLC ≥ 97.5%, batch number SS8710) were provided by Beijing Solaibao Technology Co., Ltd.

#### 2.2.3. Experimental Chemicals

Enzyme-linked immunosorbent assay (ELISA) kit: matrix metalloproteinase-2 (MMP-2, NO.JYM0019Mo), matrix metalloproteinase-9 (MMP-9, NO.JYM0737Mo), and vascular endothelial growth factor (VEGF, No. JYM0258Mo) were provided by Wuhan Genemei Biotechnology Co, Ltd; immunohistochemical reagents: goat serum blocking solution (EE0008), goat anti-rabbit IgG (*H* + *L*) HRP (EF0002), endogenous peroxidase blocking solution (EE0007), sodium citrate antigen retrieval solution (50x) (EE0005), and hematoxylin and eosin staining kit (EE0012) were provided by Shandong Sikejie Biotechnology Co, Ltd.; antibody against vascular endothelial growth factor (anti-VEGF, bs-1313R), matrix metalloproteinase-2 antibody (anti-MMP-2, bs-4605R), and matrix metalloproteinase-9 antibody (anti-MMP-9, bs-4593R) were provided by Beijing Boaosen Biotechnology Co., Ltd.

#### 2.2.4. Analysis of Baicalein, *β*-Sitosterol, and Stigmasterol with High-Performance Liquid Chromatography (HPLC) Method

Baicalein, *β*-sitosterol, and stigmasterol reference standards were accurately weighed, respectively, and ultrasonically dissolved with the appropriate amount of methanol to yield a reference substance reserve solution. The baicalein, stigmasterol, and *β*-sitosterol reference solution was measured and diluted with methanol to produce a series of standard solutions. For chromatographic separation, an Agilent Zorbax SB-C18 column (250 mm 4.6 mm, 5 m) was employed. Baicalein was analyzed at a flow rate of 1.0 mL/min using a solvent consisting of 60 : 40 (v/v) methanol and 0.4% phosphoric acid in water solution. The injection volume was set to 10 *μ*L, the column temperature to 30°C, and the detecting wavelength to 270 nm. For the analysis of stigmasterol and *β*-sitosterol, the following conditions were used: a flow rate of 1.0 mL/min, an injection volume of 10 *μ*L, a column temperature of 30°C, and methanol as the mobile phase. The detecting wavelength was 210 nm. The linear regression of peak area (*Y*) to concentration (*X*) showed that the standard curve equation of baicalein was *Y* = 46.218*X* + 0.421 (*R*^2^ = 0.9992), and the linear range of baicalein was 3.7∼370 *μ*g/mL. The standard curve equation of stigmasterol and *β*-sitosterol was *Y* = 81.364X-0.747 (*R*^2^ = 0.9997) and *Y* = 132.027*X* + 1.245 (*R*^2^ = 0.9993), respectively. The linear range of stigmasterol and *β*-sitosterol was 5.1∼510 *μ*g/mL and 2.4∼240 *μ*g/mL.

#### 2.2.5. Construction, Grouping, and Dosing

AM models were induced by tamoxifen citrate [31, 32]. Female mice and male mice were randomly paired in a 2:1 ratio [33]. And 40 female mice were randomly selected on days 2–5 after birth. 5 *μ*l/g peanut oil/lecithin/condensed milk mixture (volume ratio 2 : 0.2 : 3) was fed and 2.7 *μ*mol/kg tamoxifen was added according to body weight. Ten female mice (randomly selected) were simultaneously administered the same amount of the solvent without tamoxifen as the blank control group. After 2 months, 10 model mice were randomly selected to examine uterine tissue. The hematoxylin and eosin (HE) staining showed that the structure of the endometrium was disrupted and the glands and stromal cells had invaded the myometrium, confirming that the modeling was successful [34]. The model mice were randomly divided into the model group (*n* = 10), the mifepristone group (*n* = 10), and the GFW group (*n* = 10). In addition, 10 mice were set as the blank control group. The daily dose of mice in each treatment group was converted according to the formula equivalent dose converted by body surface area between humans and animals. The daily dose of the mifepristone group was 3.2 mg/kg, and the GFW group was 0.8 g/kg, once daily, for 30 consecutive days. After the procedure, mice were anesthetized by an intraperitoneal injection of 1% sodium pentobarbital at a bodyweight of 50 mg/kg, and blood was collected by puncture of the main abdominal vein.

#### 2.2.6. Histomorphological Observation and HE Staining

Firstly, the changes in the uterus of the mice were visually observed, whether the surface is smooth and whether nodules are present. Then, the uteri of the mice were fixed with 4% paraformaldehyde and embedded in paraffin, and the paraffin sections were stained with HE. The changes in the endometrium and myometrium of the mice in each group were observed under the microscope [35].

#### 2.2.7. ELISA Experiments

After blood collection, the blood was to stand at room temperature for 30 minutes and then centrifuged at 3000 rpm/min for 15 minutes. Serum MMP-2, MMP-9, and VEGF were determined using an ELISA kit (operation method refer to instructions manual) [36].

#### 2.2.8. Immunohistochemical Analysis

Uteri were fixed with 4% formaldehyde, embedded in paraffin, and then routinely sectioned, deparaffinized, hydrated, treated with antigen retrieval solution, and blocked with serum. The sections were incubated overnight at 4°C with anti-MMP-2 (rabbit polyclonal, 1 : 200), anti-MMP-9 (rabbit polyclonal; 1 : 200), and anti-VEGF (rabbit polyclonal; 1 : 200) primary antibodies. Then incubated with goat anti-rabbit IgG (1 : 200) secondary antibody, stained, and mounted. Under a 200x microscope, 5 images were randomly selected to observe the positive expression of MMP-2, MMP-9, and VEGF. Image Pro Plus 6.0 image analysis software (Media Cybernetics, USA) was used to analyze the images of 5 different areas in each group. The average integral optical density (AOD) was determined by measuring the covering epithelial area and the integral optical density (IOD) of positive expression in that area. The greater the AOD, the higher the degree of positive expression.

#### 2.2.9. Statistical Analysis

SPSS 21.0 (IBM Corporation, USA) and GraphPad Prism 9.0 (GraphPad Software, USA) statistical software were used for analysis and graphing. Measured data were expressed in terms of mean ± standard deviation ($\bar{x}\pm s$), and comparison between multiple groups according to the normal distribution was performed by one-way analysis of variance (one-way ANOVA). The Kruskal–Wallis H rank-sum test was used to compare multiple groups with nonnormal distribution, and *P* < 0.05 indicated that the difference was statistically significant.

## 3. Results

### 3.1. Results of Network Pharmacology

#### 3.1.1. Selection of Active Compounds and Targets

After searching the TCMSP and SymMap databases, 494 GFW compounds were tentatively identified. Under the conditions of OB ≥ 30% and DL ≥ 0.18, a total of 89 active compounds were obtained, including 8 compounds from *Cinnamon Twig*, 16 compounds from *Poria*, 11 compounds from *Cortex Moutan*, 25 compounds from *Peach Kernel*, 29 compounds from *Red Peony*. Among them, hederagenin is the common active compound of *Poria* and *Peach Kernel*; (+)-catechin and sitosterol are the common active compounds of *Cinnamon Twig*, *Cortex Moutan*, and *Red Peony*; *β*-sitosterol is the common active compound of *Cinnamon Twig*, *Peach Kernel*, and *Red Peony*. After removing duplicate targets, 102 related targets were obtained. Combining the results, sitosterol, hederagenin, *β*-sitosterol, and (+)-catechin were widely distributed and abundant, which could play a better role in treating AM. The study also provides a reference for an in-depth and detailed investigation of the GFW.

#### 3.1.2. Screening of AM Targets and Acquisition of Common Targets of GFW-AM

**Figure 2:**
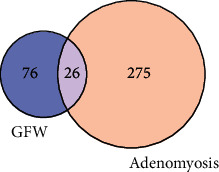
Venn diagram of GFW-AM common targets. The pink circle indicates the AM targets, the blue circle indicates the GFW target, and the intersection of the two circles is the common targets.

After searching for AM genes in GeneCards, DisGeNET, and DrugBank databases, 301 targets were identified, and the Venn diagram was constructed based on the intersection of AM and GFW targets. Finally, 26 common targets of GFW in AM were found to be potential targets for therapeutic effects (Figure 2).

#### 3.1.3. Construction of an Active Compound-Target Network of GFW and PPI Network Analysis

**Figure 3:**
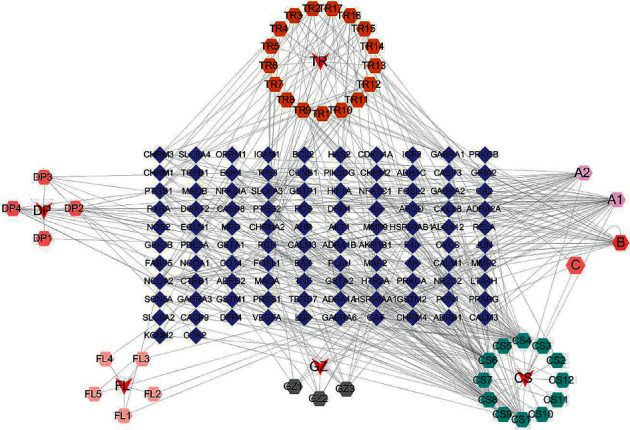
Active component-target network diagram of GFW. The red nodes represent 5 traditional Chinese drugs, the blue nodes represent the targets, and the other colors represent the compound ingredients of different drugs. A1 and A2 are the active compounds shared by *Cinnamon Twig*, *Cortex Moutan*, and *Red Peony*; B is the active compound shared by *Poria* and *Peach Kernel*; and C is the active compound of *Cinnamon Twig*, *Peach Kernel*, and *Red Peony*.

Cytoscape 3.7.2 was used to construct the network of the active compounds and targets of GFW (Figure 3). The network showed that 152 nodes were connected by 390 edges, with the red nodes representing 5 traditional Chinese drugs, the blue nodes representing the targets, and the other colors representing the active compounds of the different drugs. It was found that there was a multilevel and multidimensional correspondence relationship between the active compounds and the targets of GFW, which formed a complex network system. Among them, the active compounds with a high degree of GFW are baicalein (degree = 38), *β*-sitosterol (degree = 36), stigmasterol (degree = 28), ellagic acid (degree = 21), and (+)-catechin (degree = 15). Possibly via common targets such as AKT1 (degree = 23), VEGFA (degree = 20), MMP-9 (degree = 19), and MMP-2 (degree = 17) play a role.

**Figure 4:**
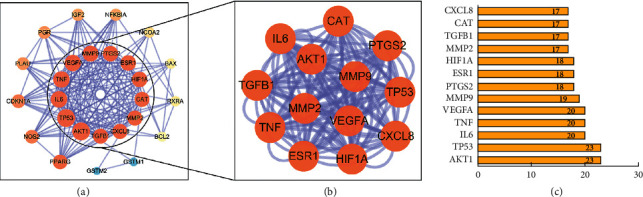
(a) PPI network of GFW-AM; (b) key targets of GFW-AM. Nodes size and color are proportional to degree values and combined scores. (c) Degree values of key targets.

To investigate the specific mechanism of GFW in treating AM, we constructed a PPI network of common targets using the STRING database and Cytoscape 3.7.2 (Figure 4(a)). Consisting of 26 nodes and 185 edges, its average node degree is 15.5, and its avg. local clustering coefficient is 0.805. Nodes with different colors reflect the degree values of distinct targets by using Cytoscape 3.7.2 to analyze the PPI of common targets. The darker the color, the greater the degree values, and the blue edges represent the interaction between the targets. AKT1, TP53, IL6, TNF, VEGFA, MMP9, PTGS2, ESR1, HIF1A, MMP2, TGFB1, CAT, and CXCL8 were key targets with degree values ≥ median (Figure 4(b)-4(c)). The 13 key targets might play a critical role in GFW treating AM.

#### 3.1.4. Results of GO and KEGG Enrichment Analyses

**Figure 5:**
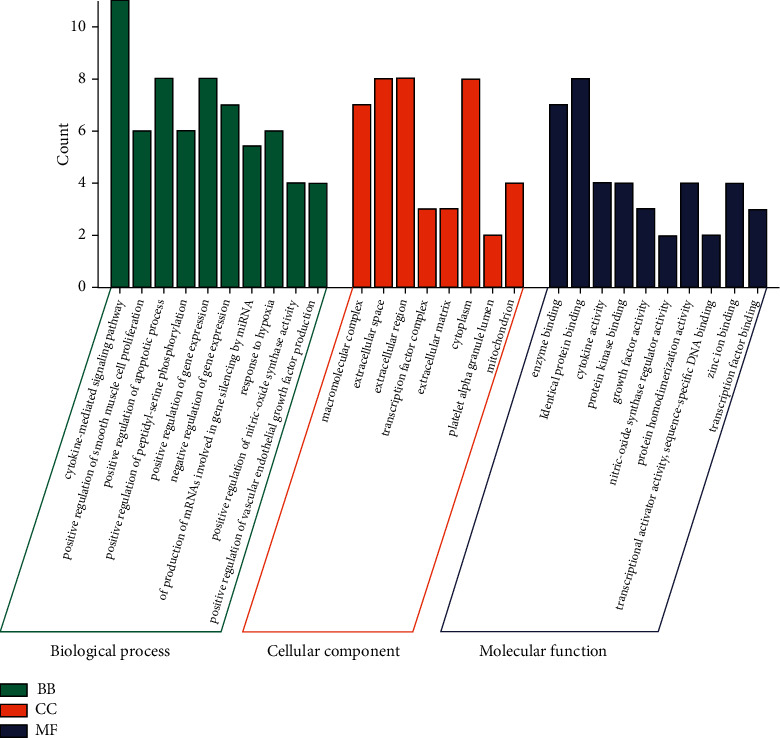
GO enrichment analysis of GFW in treating AM. Green bars represent the BP enrichment process, red bars represent the CC enrichment process, and blue bars represent the MF enrichment process.

The DAVID database was utilized to conduct GO enrichment analysis on the key targets to further explore the mechanism of GFW in treating AM. A total of 188 biological functions and 53 signaling pathways were enriched with *P* < 0.05, including 162 items in the biological processes (BP), 18 items in the molecular functions (MF), and 8 items in the cell component (CC). The top 10 GO enrichment showed were chosen for study, and the *P* values were ranked from small to large (Figure 5). The results revealed that the BP was primarily related to positive regulation of vascular endothelial cell migration, inflammatory response, and immune response during the GFW process in treating AM. The MF is mainly comprised of cytokine activity, enzyme binding, and transcription factor binding, while the CC is mostly made up of the cytoplasm and nucleus.

**Figure 6:**
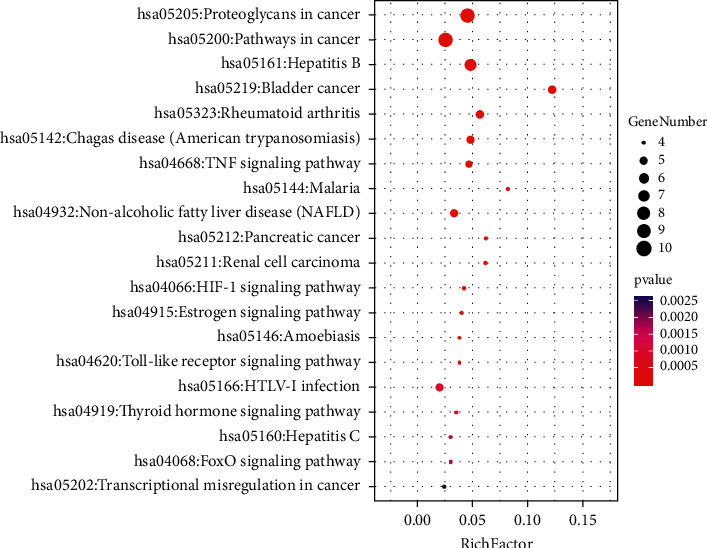
KEGG enrichment analysis of GFW in treating AM. The *x*-axis represents the enrichment factors. The *y*-axis represents the pathway names. The size of the bubbles represents the number of genes; the larger the bubble, the more the number of genes. The color of the bubbles represents the *P* value; the redder the color, the smaller the *P* value.

KEGG enrichment analysis contained 53 signaling pathways. The top 20 signaling pathways were chosen to show the KEGG enrichment analysis through the bubble chart based on the size of the *P* value (Figure 6). The results revealed that the Toll-like receptor signaling pathway, the tumor necrosis factor (TNF) signaling pathway, and hypoxia-inducible factor-1 (HIF-1) were all related to the GFW in treating AM.

#### 3.1.5. Molecular Docking Verification

**Figure 7:**
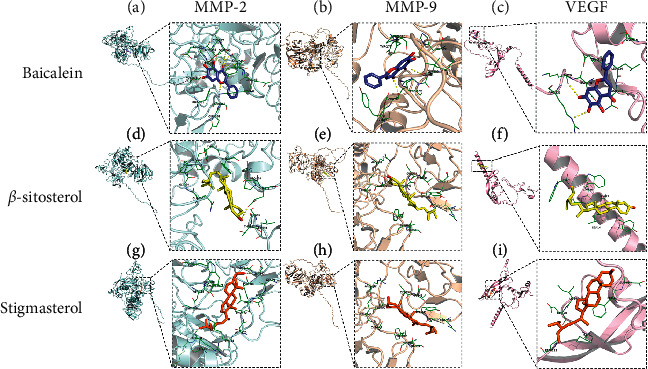
The 3D diagrams of the docking. (a). Baicalein with MMP-2; (b) baicalein with MMP-9; (c) baicalein with VEGF; (d) *β*-sitosterol with MMP-2; (e) *β*-sitosterol with MMP-9; (f) *β*-sitosterol with VEGF; (g) stigmasterol with MMP-2; (h) stigmasterol with MMP-9; and (i) stigmasterol with VEGF.

Because the pathogenesis of AM is related to ectopic intimal invasion, metastasis, and abnormal angiogenesis, studies indicated that MMP-2, MMP-9, and VEGF were the primary targets involved in ectopic lesion invasion and metastasis [37]. As a result, baicalein (degree = 38), *β*-sitosterol (degree = 36), and stigmasterol (degree = 28) were chosen as the top 3 components in GFW based on the degree values. MMP-2, MMP-9, and VEGF, involved in invasion and metastasis, were selected for molecular docking to verify the tightness of the binding between the active compounds and the targets. AutoDock Vina performed the molecular docking. The higher the absolute values of the docking results, the greater the probability of playing a role. The absolute values of the binding energies of baicalein, *β*-sitosterol, and stigmasterol to MMP-2, MMP-9, and VEGF were all greater than 5.0 kcal/mol, according to molecular docking results.  Table 1 shows the unique docking binding energies, and Figure 7 depicts the 3D diagrams of the docking. It can be noted that active compounds like baicalein, *β*-sitosterol, and stigmasterol, and targets like MMP-2, MMP-9, and VEGF may be important to prevent migration and invasion.

### 3.2. Experimental Results

Despite its benign nature, AM has the ability to migrate and invade in a manner similar to malignant tumors. MMP-2, MMP-9, and VEGF are all involved in AM invasion and migration [37]. The results of network pharmacology indicated that the key targets of GFW in the therapy of AM were MMP-2, MMP-9, and VEGF, all of which were involved in cell invasion, migration, and angiogenesis. GFW had been proven in the study to decrease matrix metalloproteinase (MMP) levels in endometriosis (EM) rats and to limit the invasion and migration of ectopic endometrium [38]. However, no relevant research based on network pharmacology and experimental verification demonstrates that GFW inhibits ectopic endometrial invasion and migration in AM. Additionally, network pharmacology prediction has several drawbacks. To validate the accuracy of network pharmacology, MMP-2, MMP-9, and VEGF were chosen for in vivo verification because they were all associated with invasion and migration among the key targets.

#### 3.2.1. Determination of Baicalein, *β*-Sitosterol, and Stigmasterol by HPLC

In the chromatogram, baicalein, *β*-sitosterol, and stigmasterol were all baseline separated from other ingredient. The contents of those components are baicalein 5.103 ± 0.12 mg/g, stigmasterol 1.867 ± 0.09 mg/g, and *β*-sitosterol 2.014 ± 0.21 mg/g (Figure S1).

#### 3.2.2. Histomorphological Observation

**Figure 8:**
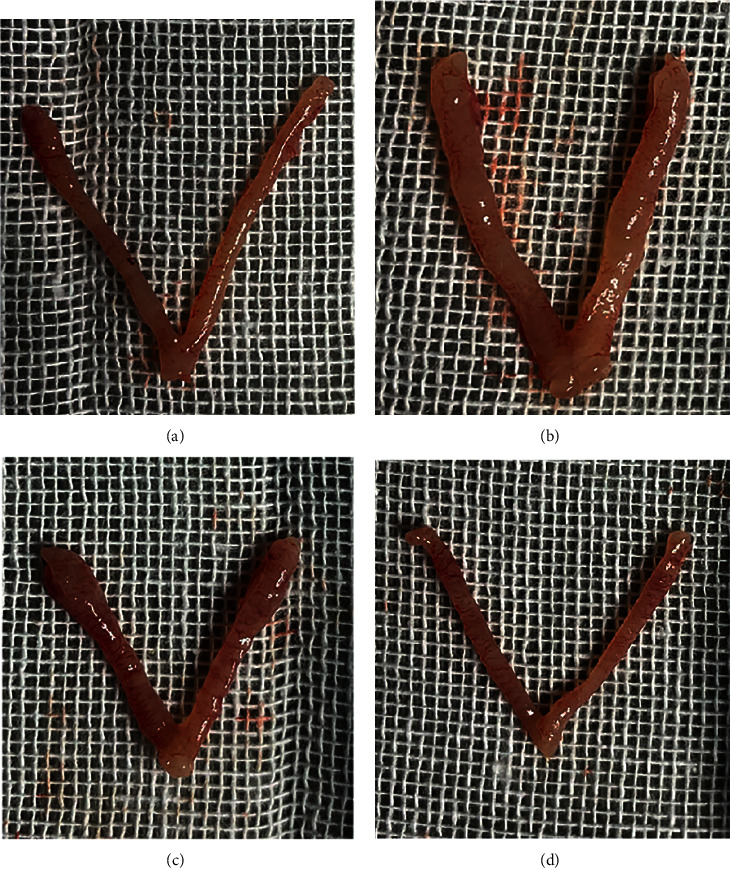
Histomorphological observation of AM mice uteri in each group after 30 days of intervention. (a) Blank control group; (b) model group; (c) mifepristone group; (d) GFW group.

After 30 days of intervention, uteri were taken from the mice (Figure 8). The uteri in the blank control group were light red, smooth, and free of apparent nodules (Figure 8(a)); those in the model group had varying degrees of uterine congestion, thickness, local swelling, and nodules (Figure 8(b)). The uteri in the mifepristone group were somewhat thickened but had no visible nodules (Figure 8(c)); the uteri in the GFW had no obvious thickening and nodules (Figure 8(d)).

#### 3.2.3. HE Staining

**Figure 9:**
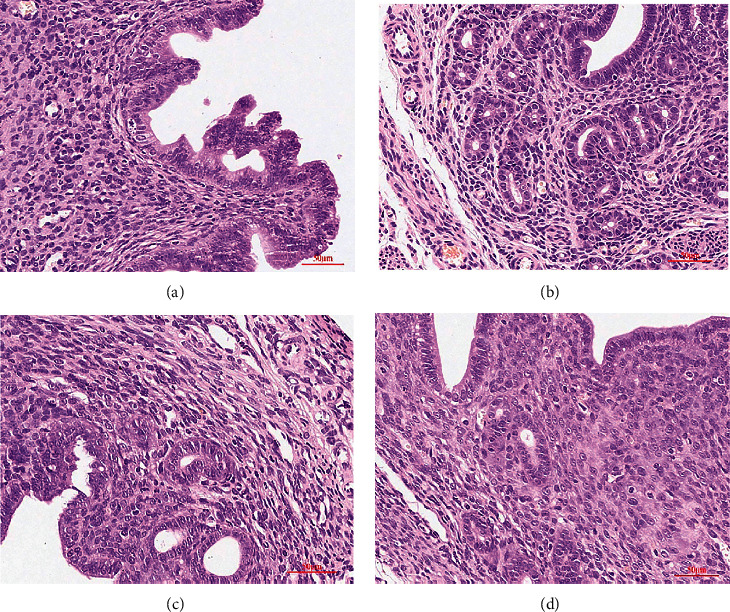
HE staining of AM mice uteri in each group after 30 days of intervention (HE 200x). (a) Blank control group; (b) model group; (c) mifepristone group; and (d) GFW group.

Figure 9 showed the results of HE staining. The endometrial structure of the blank control group was normal, the endometrium-myometrium boundary was visible, although the myometrium had a small number of glands and stromal cells (Figure 9(a)). The disordered myometrium-endometrial structure boundary vanished in the model group, and endometrial glands and interstitial cells invaded the myometrium (Figure 9(b)). There were glands, interstitial cells, and endometrium in the local myometrium of the mifepristone group, but they were less than those in the model group (Figure 9(c)). The GFW group had significantly fewer endometrial and myometrial lesions than the model group, the myometrium had fewer glands, stromal cells, and endometrium than the model group (Figure 9(d)).

#### 3.2.4. Comparison of Serum Levels of MMP-2, MMP-9, and VEGF in Each Group

The treatment of AM with GFW is closely associated with invasion, migration, and angiogenesis, according to the results of network pharmacology in this study. To assess the therapeutic impact, the serum MMP-2, MMP-9, and VEGF levels in each group were examined (Table 2). In the model group, the levels of MMP-2, MMP-9, and VEGF were significantly higher than in the blank control group (^*∗*^*P* < 0.05). Compared with the model group, mifepristone and GFW groups were reduced to varying degrees (^#^*P* < 0.05), and the GFW group was better than the mifepristone group in reducing MMP-2, MMP-9, and VEGF levels (^★^*P* < 0.05). GFW appeared to be crucial in preventing the invasion, metastasis, and angiogenesis of AM lesions.

#### 3.2.5. Comparison of MMP-2, MMP-9, and VEGF Protein Expression in Each Group

**Figure 10:**
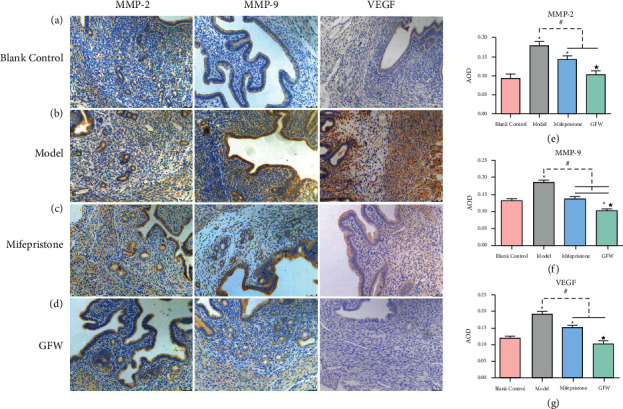
Immunohistochemical staining of MMP-2, MMP-9, and VEGF (IHC 200x). (a) Blank control group; (b) model group; (c) mifepristone group; (d) GFW group; (e) statistical bar chart of MMP-2 expression in each group; (f) statistical bar chart of MMP-9 expression in each group; and (g) statistical bar chart of VEGF expression in each group. Compared with the blank control group, ^*∗*^*P* < 0.05; compared with the model group, ^#^*P* < 0.05; compared with the mifepristone group, ^★^*P* < 0.05.

The immunohistochemistry staining of the uteri in each group is shown in Figures 10(a)–10(d). The positive expression in the model group was higher. In comparison, the expression in the blank control group, mifepristone group, and GFW group was lower, indicating that the model group had higher MMP-2, MMP-9, and VEGF content, whereas the mifepristone and GFW groups had lower content. MMP-2, MMP-9, and VEGF are depicted statistically in Figures 10(e)–10(g). The positive expression of MMP-2, MMP-9, and VEGF in the model group was much higher than in the blank control group (^*∗*^*P* < 0.05). The GFW group and mifepristone significantly reduced MMP-2, MMP-9, and VEGF levels compared to the model group (^#^*P* < 0.05). Additionally, the GFW group demonstrated a substantial advantage over the mifepristone group (^★^*P* < 0.05).

## 4. Discussion

The famous Chinese classical prescription GFW created by Zhang Zhongjing in the Eastern Han Dynasty is mainly used to treat blood stasis diseases. Clinical studies found that GFW could reduce the degree of dysmenorrhea in patients with AM and decrease the amount of menstruation [39]. Zhang indicated that GFW reduced the level of VEGF in EM, inhibited the invasion and migration by inhibiting the growth of blood vessels in ectopic lesions [40], and also played anti-inflammatory and endocrine-regulating effects [41]. However, less research has been done on AM. Mori found that GFW decreased the levels of interleukin 6 (IL-6), interleukin 8 (IL-8), and TNF-*α* in peritoneal fluid; reduced the number of macrophages; and then inhibited the growth of ectopic endometrial neovascularization. The reduction in the formation of new blood vessels also inhibited the levels of MMP-2 and MMP-9 in vivo, further preventing the invasion and adhesion of ectopic cells [36]. Our research demonstrated that GFW decreased the expression of MMP-2, MMP-9, and VEGF and had an inhibitory effect on the invasion and migration of AM, which was consistent with the conclusions of published studies.

AM is a benign gynecological disease, but it has tumor-like invasive and metastatic properties. Multiple uterine cavity surgeries damage the ultrastructure of the endometrium-myometrium and change the morphology of mast cells; furthermore, the myometrium is invaded by active eutopic endometrial glands and stroma. In this process, the degradation of extracellular matrix (ECM) and the formation of new blood vessels exacerbate the progression of AM, in which matrix metalloproteinases (MMPs) are involved. Although the MMP family has many members, MMP-2 and MMP-9 have great potential to enhance cell migration and invasion ability as important Zn^2+^-dependent matrix metalloproteinases [42, 43]; in addition, they are highly expressed in patients with AM and EM [44, 45]. MMP-2 and MMP-9 are more critical for angiogenesis than for ECM degradation, which are considered to be the key to promoting tumor angiogenesis. VEGF can promote the growth of vascular endothelial cells so that the ectopic endometrium can form new capillary loops during implantation and transfer [37, 46]. Li et al. showed that the expression of MMP-2 and MMP-9 was enhanced in AM and positively correlated with the production of VEGF, indicating that MMP-2 and MMP-9 aggravated the invasion and metastasis of AM by degrading ECM and promoting angiogenesis [37]. Thus, complex biological processes such as cell invasion and metastasis induced by MMP-2 and MMP-9, as well as angiogenesis and metastasis involving VEGF, are the essential pathological basis for the progression of ectopic AM lesions.

In the network pharmacology study, baicalein, sitosterol, *β*-sitosterol, (+)-catechin, and other active compounds inhibited the invasion and metastasis of AM ectopic lesions, possibly by acting on MMP-2, MMP-9, and VEGF. Some mechanisms have been confirmed, (+)-catechin decreased VEGF levels in vivo and inhibited the growth of ectopic endometrium [47]. Stigmasterol can inhibit angiogenesis, reduce the invasive ability of tumor cells, promote the apoptosis of abnormal cells, and improve the immunity of the body [48]. GO and KEGG enrichment analyses revealed that biological processes such as vascular endothelial migration, cell proliferation, and inflammatory responses and signaling pathways such as TNF and HIF-1 were closely related to GFW treatment of AM. The TNF signaling pathway induced the production of VEGF by activating nuclear factor kappa-B (NF-*κ*B), thereby promoting the formation of new blood vessels in AM and increasing the invasiveness of ectopic cells [49, 50]. Xue et al. found that HIF-1 promoted the formation of new blood vessels in ectopic tissues under the environment of inflammation and hypoxia, thereby regulating the invasion and metastasis of ectopic endometrium [51, 52]. In addition, GFW reduced the expression of VEGF by downregulating the level of HIF-1*α* in patients with EM [53]. In summary, it was speculated that GFW reduced the level of VEGF by regulating TNF, HIF-1, and other signaling pathways, thereby inhibiting AM angiogenesis as well as the invasion and metastasis of ectopic cells. Molecular docking was performed to verify the tightness of the binding between the key compounds of GFW and key targets MMP-2, MMP-9, and VEGF. Based on the network pharmacology research and literature verification, we speculated that GFW played a therapeutic role in AM by inhibiting angiogenesis and decreasing cell invasion ability in multiple levels and dimensions.

Network pharmacology might have false-positive results in the prediction process due to the disadvantages of irregular data extraction, a large amount of data, and a lack of unified screening standards. Therefore, high-quality and high-evidence verification results need to be obtained through further experimental research while improving the accuracy of the data.

Based on the mechanisms of AM invasion, metastasis, and angiogenesis, animal experiments were performed to verify the accuracy of network pharmacology. This study used the recognized AM modeling method in ICR mice. The targets MMP-2, MMP-9, and VEGF, which were mainly involved in AM invasion, metastasis, and angiogenesis, were detected by ELISA and immunohistochemistry. The results confirmed that the expression of MMP-2, MMP-9, and VEGF increased during the progression of AM. We believed that this was closely related to the invasion, metastasis, and angiogenesis of AM lesions. GFW drastically reduced the expression of MMP-2, MMP-9, and VEGF, indicating that GFW can inhibit the invasion, metastasis, and angiogenesis of AM lesions, which was consistent with the previous network pharmacology prediction results. However, further experiments with large samples are still needed to verify the accuracy of the results due to the small sample size of this study, different responses of the individual mouse to drugs, and differences in the choice of experimental reagents.

In summary, various compounds in GFW might play a role in treating AM through key targets such as MMP-2, MMP-9, and VEGF. The findings of this study provided a basis for studying the mechanism of action of GFW in treating AM. However, obtaining complete and accurate data by relying on the network was difficult due to the complexity of TCM compounds and the uncertainty of disease targets. Therefore, the study experimentally verified the inhibitory effect of GFW on MMP-2, MMP-9, and VEGF in the invasion and metastasis of AM ectopic cells, to avert the limitations of network pharmacology. The study also provided a molecular basis for further research on the pathogenesis of AM and the therapeutic effect of GFW. Because of the limited number of targets, it was difficult to explain the complete biological process of GFW inhibiting AM invasion and metastasis. The active compounds and pathways of GFW will be further studied in the future to make up for the insufficiency of this study and provide high-quality and high-evidence results for the mechanism of GFW in treating AM.  Table S1 shows the GFW-related compounds and targets.  Table S2 shows the AM-related targets.  Table S3 shows the GFW-AM common targets.  Table S4 shows the GFW-AM common targets' string interactions and key targets.

## 5. Conclusions

To sum up, the active compounds of GFW in treating AM mainly include baicalein, *β*-sitosterol, and stigmasterol. The key targets for inhibiting the invasion of ectopic AM are MMP-2, MMP-9, and VEGF. According to the results of animal experiments, GFW can significantly reduce the levels of MMP-2, MMP-9, and VEGF in AM and inhibit the invasion, migration, and angiogenesis of ectopic lesions, so as to achieve the purpose of treating AM. However, due to the limitation of the number of targets, the interactive therapeutic effects of GFW multi-compounds and multi-targets in the treatment process are still not fully clear. The active compounds and pathways of GFW need to be further studied in the future to make up for the insufficiency of this study and provide high-quality and high-evidence results for the mechanism of GFW in treating AM.

## Figures and Tables

**Table 1:** Minimum binding energies for key compounds and key targets.

Key targets	UniProt ID	Key compounds	Binding energy (kcal/mol.)
MMP-2	P08253	Baicalein	−7.9
MMP-9	P14780	Baicalein	−7.9
VEGF	P15692	Baicalein	−6.0
MMP-2	P08253	*β*-Sitosterol	−7.8
MMP-9	P14780	*β*-Sitosterol	−7.9
VEGF	P15692	*β*-Sitosterol	−6.3
MMP-2	P08253	Stigmasterol	−8.5
MMP-9	P14780	Stigmasterol	−8.4
VEGF	P15692	Stigmasterol	−6.6

**Table 2:** Comparison of serum levels of MMP-2, MMP-9, and VEGF in each group ($\bar{x}\pm s$, pg·mL^−1^, *n* = 5).

Group	*N*	MMP-2 (pg/mL)	MMP-9 (pg/mL)	VEGF (pg/mL)
Blank control	5	158.57 ± 4.20	216.67 ± 14.05	17.42 ± 1.53
Model	5	196.60 ± 6.05^*∗*^	345.50 ± 25.79^*∗*^	21.59 ± 0.30^*∗*^
Mifepristone	5	159.82 ± 5.79^#^	260.44 ± 6.59^*∗*^^#^	18.66 ± 0.68^#^
GFW	5	137.64 ± 5.46^*∗*^^#★^	236.89 ± 6.70^#★^	16.44 ± 0.92^#★^

Compared with the blank control group, ^*∗*^*P* < 0.05; compared with the model group, ^#^*P* < 0.05; compared with the mifepristone group, ^★^*P* < 0.05.

## Data Availability

All data of this study can be obtained from the provided open platform website. Experimental data and analyses can be provided by the corresponding authors where reasonable.

## Supplementary Materials

Supplementary Materials Figure S1: HPLC figure of baicalein, *β*-sitosterol, and stigmasterol. Table S1: GFW-related compounds and targets. Table S2: AM-related targets. Table S3: GFW-AM common targets. Table S4: GFW-AM common targets' string interactions and key targets.
