# Supplementary material for: Analysis of the Mechanism of GuizhiFuling Wan in Treating Adenomyosis Based on Network Pharmacology Combined with Molecular Docking and Experimental Verification
Source: Evid Based Complement Alternat Med. 2022 Aug 26;2022:6350257. doi: 10.1155/2022/6350257 (PMC9440632; doi:10.1155/2022/6350257)
Supplement: Supplementary Materials — Figure S1: HPLC figure of baicalein, β-sitosterol, and stigmasterol. Table S1: GFW-related compounds and targets. Table S2: AM-related targets. Table S3: GFW-AM common targets. Table S4: GFW-AM common targets' string interactions and key targets. [file 6350257.f1.zip › Supplementary Table S1.pdf]

Supplementary Table S1 GFW related compounds and targets

	Mol ID	compounds	protein names	gene names
Guizhi	MOL001736	(-)-taxifolin	Prostaglandin G/H synthase 1	PTGS1
	MOL001736	(-)-taxifolin	Prostaglandin G/H synthase 2	PTGS2
	MOL001736	(-)-taxifolin	Heat shock protein HSP 90	HSP90AB1
	MOL001736	(-)-taxifolin	Heat shock protein HSP 90	HSP90AA1
	MOL001736	(-)-taxifolin	Phosphatidylinositol-4,5-bisphosphate 3-kinase catalytic subunit, gamma isoform	
	MOL001736	(-)-taxifolin	Phosphatidylinositol-4,5-bisphosphate 3-kinase catalytic subunit, gamma isoform	
	MOL001736	(-)-taxifolin	Phosphatidylinositol-4,5-bisphosphate 3-kinase catalytic subunit, gamma isoform	
	MOL000073	ent-Epicatechin	Prostaglandin G/H synthase 1	PTGS1
	MOL000073	ent-Epicatechin	Estrogen receptor	ESR1
	MOL000073	ent-Epicatechin	Prostaglandin G/H synthase 2	PTGS2
	MOL000073	ent-Epicatechin	Heat shock protein HSP 90	HSP90AB1
	MOL000073	ent-Epicatechin	Heat shock protein HSP 90	HSP90AA1
	MOL000073	ent-Epicatechin	Beta-lactamase	
	MOL000073	ent-Epicatechin	mRNA of PKA Catalytic Subunit C-alpha	
	MOL000492	(+)-catechin	Prostaglandin G/H synthase 1	PTGS1
	MOL000492	(+)-catechin	Estrogen receptor	ESR1
	MOL000492	(+)-catechin	Estrogen receptor	ESR1
	MOL000492	(+)-catechin	Estrogen receptor	ESR1
	MOL000492	(+)-catechin	Prostaglandin G/H synthase 2	PTGS2
	MOL000492	(+)-catechin	Heat shock protein HSP 90	HSP90AB1
	MOL000492	(+)-catechin	Heat shock protein HSP 91	HSP90AA1
	MOL000492	(+)-catechin	Nuclear receptor coactivator 2	NCOA2
	MOL000492	(+)-catechin	Calmodulin	CALM1
	MOL000492	(+)-catechin	Calmodulin	CALM3
	MOL000492	(+)-catechin	Retinoic acid receptor RXR-alpha	RXRA
	MOL000492	(+)-catechin	Catalase	CAT
	MOL004576	taxifolin	Prostaglandin G/H synthase 1	PTGS1
	MOL004576	taxifolin	Prostaglandin G/H synthase 2	PTGS2
	MOL004576	taxifolin	Heat shock protein HSP 90	HSP90AB1
	MOL004576	taxifolin	Heat shock protein HSP 90	HSP90AA1
	MOL004576	taxifolin	Phosphatidylinositol-4,5-bisphosphate 3-kinase catalytic subunit, gamma isoform	
	MOL004576	taxifolin	Phosphatidylinositol-4,5-bisphos	

			phate 3-kinase catalytic subunit, gamma isoform	
	MOL004576	taxifolin	Phosphatidylinositol-4,5-bisphos phate 3-kinase catalytic subunit, gamma isoform	
	MOL004576	taxifolin	Retinoic acid receptor RXR-alpha	RXRA
	MOL004576	taxifolin	Aldose reductase	AKR1B1
	MOL004576	taxifolin	Transcription factor p65	RELA
	MOL004576	taxifolin	Metal-binding activator 1	
	MOL004576	taxifolin	Intercellular adhesion molecule 1	ICAM1
	MOL004576	taxifolin	Diacylglycerol O-acyltransferase 2	DGAT2
	MOL000359	sitosterol	Progesterone receptor	PGR
	MOL000359	sitosterol	Nuclear receptor coactivator 2	NCOA2
	MOL000359	sitosterol	Mineralocorticoid receptor	NR3C2
	MOL000358	beta-sitosterol	Progesterone receptor	PGR
	MOL000358	beta-sitosterol	Nuclear receptor coactivator 2	NCOA2
	MOL000358	beta-sitosterol	Prostaglandin G/H synthase 1	PTGS1
	MOL000358	beta-sitosterol	Prostaglandin G/H synthase 2	PTGS2
	MOL000358	beta-sitosterol	Heat shock protein HSP 90	HSP90AB1
	MOL000358	beta-sitosterol	Heat shock protein HSP 90	HSP90AA1
	MOL000358	beta-sitosterol	Phosphatidylinositol-4,5-bisphos phate 3-kinase catalytic subunit, gamma isoform	
	MOL000358	beta-sitosterol	Phosphatidylinositol-4,5-bisphos phate 3-kinase catalytic subunit, gamma isoform	
	MOL000358	beta-sitosterol	Phosphatidylinositol-4,5-bisphos phate 3-kinase catalytic subunit, gamma isoform	
	MOL000358	beta-sitosterol	Potassium voltage-gated channel subfamily H member 2	KCNH2
	MOL000358	beta-sitosterol	mRNA of PKA Catalytic Subunit C-alpha	
	MOL000358	beta-sitosterol	Dopamine D1 receptor	DRD1
	MOL000358	beta-sitosterol	Dopamine D1 receptor	DRD1
	MOL000358	beta-sitosterol	Muscarinic acetylcholine receptor M3	CHRM3
	MOL011169	Peroxyergosterol	N/A	
**Poria**	MOL000273	(2R)-2-[(3S,5R,10S,13R,14R,16R,17R)-3,16-dihydroxy-4,4,10,13,14-pentamethyl-2,3,5,6,12,15,16,17-octahydro-1H-cyclopenta[	Mineralocorticoid receptor	NR3C2

	a]phenanthren-17-yl]-6-methylhept-5-enoic acid		
MOL000273	(2R)-2-[(3S,5R,10S,13R,14R,16R,17R)-3,16-dihydroxy-4,4,10,13,14-pentamethyl-2,3,5,6,12,15,16,17-octahydro-1H-cyclopenta[a]phenanthren-17-yl]-6-methylhept-6-enoic acid	Nuclear receptor coactivator 2	NCOA2
MOL000275	trametenolic acid	Mineralocorticoid receptor	NR3C2
MOL000276	7,9(11)-dehydropachymic acid	N/A	
MOL000279	Cerevisterol	Mineralocorticoid receptor	NR3C2
MOL000280	(2R)-2-[(3S,5R,10S,13R,14R,16R,17R)-3,16-dihydroxy-4,4,10,13,14-pentamethyl-2,3,5,6,12,15,16,17-octahydro-1H-cyclopenta[a]phenanthren-17-yl]-5-isopropyl-hex-5-enoic acid	N/A	
MOL000282	ergosta-7,22E-dien-3beta-ol	Progesterone receptor	PGR
MOL000283	Ergosterol peroxide	Progesterone receptor	PGR
MOL000285	(2R)-2-[(5R,10S,13R,14R,16R,17R)-16-hydroxy-3-keto-4,4,10,13,14-pentamethyl-1,2,5,6,12,15,16,17-octahydrocyclopenta[a]phenanthren-17-yl]-5-isopropyl-hex-5-enoic acid	N/A	
MOL000287	3beta-Hydroxy-24-methylene-8-lanostene-21-oic acid	N/A	
MOL000289	pachymic acid		
MOL000290	Poricoic acid A	N/A	
MOL000291	Poricoic acid B	N/A	
MOL000292	poricoic acid C	N/A	
MOL000296	hederagenin	Progesterone receptor	PGR
MOL000296	hederagenin	Nuclear receptor coactivator 2	NCOA2
MOL000296	hederagenin	Muscarinic acetylcholine receptor M3	CHRM3
MOL000296	hederagenin	Muscarinic acetylcholine receptor M1	CHRM1
MOL000296	hederagenin	Gamma-aminobutyric-acid receptor alpha-2 subunit	GABRA2
MOL000296	hederagenin	Gamma-aminobutyric-acid receptor alpha-3 subunit	GABRA3
MOL000296	hederagenin	Muscarinic acetylcholine receptor M2	CHRM2
MOL000296	hederagenin	Alpha-1B adrenergic receptor	ADRA1B

	MOL000296	hederagenin	Gamma-aminobutyric acid receptor subunit alpha-1	GABRA1
	MOL000296	hederagenin	Glutamate receptor 2	GRIA2
	MOL000300	dehydroeburicoic acid	N/A	
**Moutan Bark**	MOL001925	paeoniflorin_qt	N/A	
	MOL000211	Mairin	Progesterone receptor	PGR
	MOL000359	sitosterol	Progesterone receptor	PGR
	MOL000359	sitosterol	Mineralocorticoid receptor	NR3C2
	MOL000359	sitosterol	Nuclear receptor coactivator 2	NCOA2
	MOL000422	kaempferol	Dipeptidyl peptidase IV	DPP4
	MOL000422	kaempferol	Nitric oxide synthase, inducible	NOS2
	MOL000422	kaempferol	Prostaglandin G/H synthase 1	PTGS1
	MOL000422	kaempferol	Androgen receptor	AR
	MOL000422	kaempferol	Peroxisome proliferator activated receptor gamma	PPARG
	MOL000422	kaempferol	Prostaglandin G/H synthase 2	PTGS2
	MOL000422	kaempferol	Heat shock protein HSP 90	HSP90AB1
	MOL000422	kaempferol	Heat shock protein HSP 90	HSP90AA1
	MOL000422	kaempferol	Phosphatidylinositol-4,5-bisphosphate 3-kinase catalytic subunit, gamma isoform	
	MOL000422	kaempferol	mRNA of PKA Catalytic Subunit C-alpha	
	MOL000422	kaempferol	Nuclear receptor coactivator 2	NCOA2
	MOL000492	(+)-catechin	Prostaglandin G/H synthase 1	PTGS1
	MOL000492	(+)-catechin	Estrogen receptor	ESR1
	MOL000492	(+)-catechin	Prostaglandin G/H synthase 2	PTGS2
	MOL000492	(+)-catechin	Heat shock protein HSP 90	HSP90AB1
	MOL000492	(+)-catechin	Heat shock protein HSP 90	HSP90AA1
	MOL000492	(+)-catechin	Beta-lactamase	
	MOL000492	(+)-catechin	mRNA of PKA Catalytic Subunit C-alpha	
	MOL000492	(+)-catechin	Nuclear receptor coactivator 2	NCOA2
	MOL000492	(+)-catechin	Calmodulin	CALM1
	MOL000492	(+)-catechin	Calmodulin	CALM3
	MOL000492	(+)-catechin	Retinoic acid receptor RXR-alpha	RXRA
	MOL000492	(+)-catechin	Catalase	CAT
	MOL007003	benzoyl paeoniflorin	N/A	
	MOL007369	4-O-methylpaeoniflorin_qt	N/A	
	MOL007374	5-[[5-(4-methoxyphenyl)-2-furyl]methylene]barbituric acid	Estrogen receptor	ESR1
	MOL007374	5-[[5-(4-methoxyphenyl)-2-furyl	Glycogen synthase kinase-3 beta	GSK3B

		]methylene]barbituric acid		
	MOL007374	5-[[5-(4-methoxyphenyl)-2-furyl]methylene]barbituric acid	Heat shock protein HSP 90	HSP90AB1
	MOL007374	5-[[5-(4-methoxyphenyl)-2-furyl]methylene]barbituric acid		HSP90AA1
	MOL007374	5-[[5-(4-methoxyphenyl)-2-furyl]methylene]barbituric acid	Cell division protein kinase 2	CDK2
	MOL007374	5-[[5-(4-methoxyphenyl)-2-furyl]methylene]barbituric acid	mRNA of PKA Catalytic Subunit C-alpha	
	MOL007382	mudanpioside-h_qt 2	N/A	
	MOL007384	paeonidanin_qt	N/A	
	MOL000098	quercetin	Prostaglandin G/H synthase 1	PTGS1
	MOL000098	quercetin	Androgen receptor	AR
	MOL000098	quercetin	Peroxisome proliferator activated receptor gamma	PPARG
	MOL000098	quercetin	Prostaglandin G/H synthase 2	PTGS2
	MOL000098	quercetin	Heat shock protein HSP 90	HSP90AB1
	MOL000098	quercetin		HSP90AA1
	MOL000098	quercetin	Phosphatidylinositol-4,5-bisphos phate 3-kinase catalytic subunit, gamma isoform	
	MOL000098	quercetin	Nuclear receptor coactivator 2	NCOA2
	MOL000098	quercetin	Dipeptidyl peptidase IV	DPP4
	MOL000098	quercetin	Aldose reductase	AKR1B1
	MOL000098	quercetin	Trypsin-1	PRSS1
**Peach Kernel**	MOL001323	Sitosterol alpha1	Progesterone receptor	PGR
	MOL001323	Sitosterol alpha1	Prostaglandin G/H synthase 2	PTGS2
	MOL001323	Sitosterol alpha1	Gamma-aminobutyric acid receptor subunit alpha-1	GABRA1
	MOL001323	Sitosterol alpha1	Alcohol dehydrogenase 1C	ADH1C
	MOL001323	Sitosterol alpha1	Cytochrome P450-cam	
	MOL001323	Sitosterol alpha1	Mineralocorticoid receptor	NR3C2
	MOL001328	2,3-didehydro GA70	Prostaglandin G/H synthase 1	PTGS1
	MOL001328	2,3-didehydro GA70	Muscarinic acetylcholine receptor M1	CHRM1
	MOL001328	2,3-didehydro GA70	Prostaglandin G/H synthase 2	PTGS2
	MOL001328	2,3-didehydro GA70	Sodium-dependent noradrenaline transporter	SLC6A2
	MOL001328	2,3-didehydro GA70	Gamma-aminobutyric acid receptor subunit alpha-1	GABRA1
	MOL001328	2,3-didehydro GA70	Trypsin-1	PRSS1
	MOL001328	2,3-didehydro GA70	Glutamate receptor 2	GRIA2
	MOL001329	2,3-didehydro GA77	Prostaglandin G/H synthase 2	PTGS2

MOL001329	2,3-didehydro GA77	Carbonic anhydrase II	CA2
MOL001329	2,3-didehydro GA77	Gamma-aminobutyric acid receptor subunit alpha-1	GABRA1
MOL001329	2,3-didehydro GA77	Nuclear receptor coactivator 2	NCOA2
MOL001329	2,3-didehydro GA77	Gamma-aminobutyric-acid receptor subunit alpha-6	GABRA6
MOL001339	GA119	Cytochrome P450-cam	
MOL001340	GA120	Muscarinic acetylcholine receptor M3	CHRM3
MOL001340	GA120	Muscarinic acetylcholine receptor M1	CHRM1
MOL001340	GA120	Prostaglandin G/H synthase 2	PTGS2
MOL001340	GA120	Gamma-aminobutyric-acid receptor alpha-3 subunit	
MOL001340	GA120	Muscarinic acetylcholine receptor M2	CHRM2
MOL001340	GA120	Gamma-aminobutyric acid receptor subunit alpha-1	GABRA1
MOL001340	GA120	Neuronal acetylcholine receptor protein, alpha-7 chain	
MOL001340	GA120	Gamma-aminobutyric-acid receptor subunit alpha-6	
MOL001342	GA121-isolactone	Progesterone receptor	PGR
MOL001343	GA122	N/A	
MOL001344	GA122-isolactone	Progesterone receptor	PGR
MOL001348	gibberellin 17	N/A	
MOL001349	4a-formyl-7alpha-hydroxy-1-methyl-8-methylidene-4aalpha,4bbeta-gibbane-1alpha,10beta-dicarboxylic acid	Mineralocorticoid receptor	NR3C2
MOL001349	4a-formyl-7alpha-hydroxy-1-methyl-8-methylidene-4aalpha,4bbeta-gibbane-1alpha,11beta-dicarboxylic acid	Progesterone receptor	PGR
MOL001350	GA30	N/A	
MOL001351	Gibberellin A44	Mineralocorticoid receptor	NR3C2
MOL001351	Gibberellin A44	Gamma-aminobutyric acid receptor subunit alpha-1	GABRA1
MOL001351	Gibberellin A44	Gamma-aminobutyric-acid receptor subunit alpha-6	
MOL001352	GA54	Coagulation factor Xa	F10
MOL001352	GA54	Prostaglandin G/H synthase 2	PTGS2
MOL001352	GA54	mRNA of Protein-tyrosine phosphatase, non-receptor type 1	

MOL001352	GA54	Heat shock protein HSP 90	HSP90AA1
MOL001352	GA54	Heat shock protein HSP 90	HSP90AB1
MOL001352	GA54	Nuclear receptor coactivator 2	NCOA2
MOL001352	GA54	Calmodulin	CALM1
MOL001352	GA54	Calmodulin	CALM3
MOL001353	GA60	Gamma-aminobutyric-acid receptor alpha-2 subunit	GABRA2
MOL001353	GA60	Gamma-aminobutyric-acid receptor alpha-3 subunit	GABRA3
MOL001353	GA60	Muscarinic acetylcholine receptor M2	CHRM2
MOL001353	GA60	Gamma-aminobutyric acid receptor subunit alpha-1	GABRA1
MOL001353	GA60	Glutamate receptor 2	GRIA2
MOL001355	GA63	Prostaglandin G/H synthase 2	PTGS2
MOL001355	GA63	Gamma-aminobutyric acid receptor subunit alpha-1	GABRA1
MOL001355	GA63	Neuronal acetylcholine receptor protein, alpha-7 chain	GRIA2
MOL001355	GA63	Glutamate receptor 2	
MOL001355	GA63	Gamma-aminobutyric-acid receptor subunit alpha-6	
MOL001358	gibberellin 7	Muscarinic acetylcholine receptor M3	CHRM3
MOL001358	gibberellin 7	Muscarinic acetylcholine receptor M1	CHRM1
MOL001358	gibberellin 7	Prostaglandin G/H synthase 2	PTGS2
MOL001358	gibberellin 7	CGMP-inhibited 3',5'-cyclic phosphodiesterase A	PDE3A
MOL001358	gibberellin 7	Sodium-dependent dopamine transporter	SLC6A3
MOL001358	gibberellin 7	Beta-2 adrenergic receptor	ADRB2
MOL001358	gibberellin 7	Sodium-dependent serotonin transporter	SLC6A4
MOL001360	GA77	Gamma-aminobutyric-acid receptor alpha-2 subunit	GABRA2
MOL001360	GA77	Gamma-aminobutyric acid receptor subunit alpha-1	GABRA1
MOL001360	GA77	Cytochrome P450-cam	
MOL001360	GA77	Glutamate receptor 2	GRIA2
MOL001361	GA87	Prostaglandin G/H synthase 2	PTGS2
MOL001361	GA87	Carbonic anhydrase II	CA2
MOL001368	3-O-p-coumaroylquinic acid	Prostaglandin G/H synthase 1	PTGS1
MOL001368	3-O-p-coumaroylquinic acid	Prostaglandin G/H synthase 2	PTGS2

MOL001368	3-O-p-coumaroylquinic acid	mRNA of Protein-tyrosine phosphatase, non-receptor type 1	
MOL001368	3-O-p-coumaroylquinic acid	Heat shock protein HSP 90	HSP90AA1
MOL001368	3-O-p-coumaroylquinic acid	Heat shock protein HSP 90	HSP90AB1
MOL001368	3-O-p-coumaroylquinic acid	Phosphatidylinositol-4,5-bisphosphate 3-kinase catalytic subunit, gamma isoform	PIK3CG
MOL001368	3-O-p-coumaroylquinic acid	mRNA of PKA Catalytic Subunit C-alpha	
MOL001368	3-O-p-coumaroylquinic acid	Nuclear receptor coactivator 2	NCOA2
MOL001368	3-O-p-coumaroylquinic acid	Calmodulin	CALM1
MOL001368	3-O-p-coumaroylquinic acid	Calmodulin	CALM3
MOL001371	Populoside_qt	N/A	
MOL000296	hederagenin	Progesterone receptor	PGR
MOL000296	hederagenin	Nuclear receptor coactivator 2	NCOA2
MOL000296	hederagenin	Muscarinic acetylcholine receptor M3	CHRM3
MOL000296	hederagenin	Muscarinic acetylcholine receptor M1	CHRM1
MOL000296	hederagenin	Gamma-aminobutyric-acid receptor alpha-2 subunit	GABRA2
MOL000296	hederagenin	Gamma-aminobutyric-acid receptor alpha-3 subunit	GABRA3
MOL000296	hederagenin	Muscarinic acetylcholine receptor M2	CHRM2
MOL000296	hederagenin	Alpha-1B adrenergic receptor	ADRA1B
MOL000296	hederagenin	Gamma-aminobutyric acid receptor subunit alpha-1	GABRA1
MOL000296	hederagenin	Glutamate receptor 2	GRIA2
MOL000358	beta-sitosterol	Progesterone receptor	PGR
MOL000358	beta-sitosterol	Prostaglandin G/H synthase 1	PTGS1
MOL000358	beta-sitosterol	Prostaglandin G/H synthase 2	PTGS2
MOL000358	beta-sitosterol	Heat shock protein HSP 90	HSP90AA1
MOL000358	beta-sitosterol	Heat shock protein HSP 90	HSP90AB1
MOL000358	beta-sitosterol	Phosphatidylinositol-4,5-bisphosphate 3-kinase catalytic subunit, gamma isoform	PIK3CG
MOL000358	beta-sitosterol	Potassium voltage-gated channel subfamily H member 2	KCNH2
MOL000358	beta-sitosterol	mRNA of PKA Catalytic Subunit C-alpha	
MOL000358	beta-sitosterol	Dopamine D1 receptor	DRD1
MOL000358	beta-sitosterol	Muscarinic acetylcholine receptor M3	CHRM3

	MOL000493	campesterol	Progesterone receptor	PGR
	MOL000493	campesterol	Nuclear receptor coactivator 2	NCOA2
	MOL000493	campesterol	Prostaglandin G/H synthase 1	PTGS1
	MOL000493	campesterol	Prostaglandin G/H synthase 2	PTGS2
	MOL000493	campesterol	Heat shock protein HSP 90	HSP90AA1
	MOL000493	campesterol	Heat shock protein HSP 90	HSP90AB1
	MOL000493	campesterol	Phosphatidylinositol-4,5-bisphosphate 3-kinase catalytic subunit, gamma isoform	PIK3CG
	MOL000493	campesterol	Nuclear receptor coactivator 2	NCOA2
**Red Peony**	MOL001002	ellagic acid	Cell division protein kinase 2	CDK2
	MOL001002	ellagic acid	Estrogen receptor	ESR1
	MOL001002	ellagic acid	Estrogen receptor	ESR1
	MOL001002	ellagic acid	Androgen receptor	AR
	MOL001002	ellagic acid	Progesterone receptor	PGR
	MOL001002	ellagic acid	Heat shock protein HSP 90	HSP90AA1
	MOL001002	ellagic acid	Heat shock protein HSP 90	HSP90AB1
	MOL001002	ellagic acid	Transcription factor p65	RELA
	MOL001002	ellagic acid	Vascular endothelial growth factor A	VEGFA
	MOL001002	ellagic acid	Cyclin-dependent kinase inhibitor 1	CDKN1A
	MOL001002	ellagic acid	72 kDa type IV collagenase	MMP2
	MOL001002	ellagic acid	Matrix metalloproteinase-9	MMP9
	MOL001002	ellagic acid	NF-kappa-B inhibitor alpha	NFKBIA
	MOL001002	ellagic acid	Interleukin-8	CXCL8
	MOL001002	ellagic acid	Protein kinase C beta type	PRKCB
	MOL001002	ellagic acid	Glutathione S-transferase P	GSTP1
	MOL001002	ellagic acid	Glutathione S-transferase P	GSTP1
	MOL001002	ellagic acid	Glutathione S-transferase P	GSTP1
	MOL001002	ellagic acid	Insulin-like growth factor II	IGF2
	MOL001002	ellagic acid	Glutathione S-transferase Mu 1	GSTM1
	MOL001002	ellagic acid	Glutathione S-transferase Mu 2	GSTM2
	MOL001002	ellagic acid	Glutathione S-transferase A1	GSTA1
	MOL001002	ellagic acid	Glutathione S-transferase A2	GSTA2
	MOL001002	ellagic acid	Chitin synthase 2	
	MOL001918	paeoniflorgenone	Gamma-aminobutyric acid receptor subunit alpha-1	GABRA1
	MOL001921	Lactiflorin	N/A	
	MOL001924	paeoniflorin	Tumor necrosis factor	TNF
	MOL001924	paeoniflorin	Interleukin-6	IL6
	MOL001924	paeoniflorin	Monocyte differentiation antigen CD14	CD14

MOL001924	paeoniflorin	Lipopolysaccharide-binding protein	LBP
MOL001925	paeoniflorin_qt	N/A	
MOL002714	baicalein	Prostaglandin G/H synthase 1	PTGS1
MOL002714	baicalein	Androgen receptor	AR
MOL002714	baicalein	Prostaglandin G/H synthase 2	PTGS2
MOL002714	baicalein	Heat shock protein HSP 90	HSP90AA1
MOL002714	baicalein	Heat shock protein HSP 90	HSP90AB1
MOL002714	baicalein	mRNA of PKA Catalytic Subunit C-alpha	
MOL002714	baicalein	Dipeptidyl peptidase IV	DPP4
MOL002714	baicalein	Phosphatidylinositol-4,5-bisphos phate 3-kinase catalytic subunit, gamma isoform	PRSS1
MOL002714	baicalein	CGMP-inhibited 3',5'-cyclic phosphodiesterase A	PDE3A
MOL002714	baicalein	Trypsin-1	PRSS1
MOL002714	baicalein	Nuclear receptor coactivator 2	NCOA2
MOL002714	baicalein	Nuclear receptor coactivator 1	NCOA1
MOL002714	baicalein	Calmodulin	
MOL002714	baicalein	Transcription factor p65	RELA
MOL002714	baicalein	RAC-alpha serine/threonine-protein kinase	AKT1
MOL002714	baicalein	Vascular endothelial growth factor A	VEGFA
MOL002714	baicalein	Apoptosis regulator Bcl-2	BCL2
MOL002714	baicalein	Proto-oncogene c-Fos	FOS
MOL002714	baicalein	Apoptosis regulator BAX	BAX
MOL002714	baicalein	Matrix metalloproteinase-9	MMP9
MOL002714	baicalein	Caspase-3	CASP3
MOL002714	baicalein	Cellular tumor antigen p53	TP53
MOL002714	baicalein	Hypoxia-inducible factor 1-alpha	HIF1A
MOL002714	baicalein	Fos-related antigen 1	FOSL1
MOL002714	baicalein	Fos-related antigen 2	FOSL2
MOL002714	baicalein	Cell division control protein 2 homolog	
MOL002714	baicalein	G2/mitotic-specific cyclin-B1	CCNB1
MOL002714	baicalein	Myeloperoxidase	MPO
MOL002714	baicalein	Aryl hydrocarbon receptor	AHR
MOL002714	baicalein	Insulin-like growth factor II	IGF2
MOL002714	baicalein	Cytochrome c	CYCS
MOL002714	baicalein	Arachidonate 12-lipoxygenase, 12S-type	ALOX12
MOL002714	baicalein	Nuclear factor of activated	NFATC1

		T-cells, cytoplasmic 1	
MOL002714	baicalein	Tudor domain-containing protein 7	TDRD7
MOL002714	baicalein	Egl nine homolog 1	EGLN1
MOL002714	baicalein	NADPH oxidase 5	
MOL002714	baicalein	Fatty acid-binding protein, epidermal	FABP5
MOL002714	baicalein	Apolipoprotein D	APOD
MOL002776	Baicalin	Coagulation factor Xa	F10
MOL002776	Baicalin	mRNA of Protein-tyrosine phosphatase, non-receptor type 1	
MOL000358	beta-sitosterol	Progesterone receptor	PGR
MOL000358	beta-sitosterol	Nuclear receptor coactivator 2	NCOA2
MOL000358	beta-sitosterol	Prostaglandin G/H synthase 1	PTGS1
MOL000358	beta-sitosterol	Prostaglandin G/H synthase 2	PTGS2
MOL000358	beta-sitosterol	Heat shock protein HSP 90	HSP90AA1
MOL000358	beta-sitosterol	Heat shock protein HSP 90	HSP90AB1
MOL000358	beta-sitosterol	Phosphatidylinositol-4,5-bisphosphate 3-kinase catalytic subunit, gamma isoform	
MOL000358	beta-sitosterol	Potassium voltage-gated channel subfamily H member 2	KCNH2
MOL000358	beta-sitosterol	mRNA of PKA Catalytic Subunit C-alpha	
MOL000358	beta-sitosterol	Dopamine D1 receptor	DRD1
MOL000358	beta-sitosterol	Muscarinic acetylcholine receptor M3	CHRM3
MOL000358	beta-sitosterol	Muscarinic acetylcholine receptor M1	CHRM1
MOL000358	beta-sitosterol	Sodium channel protein type 5 subunit alpha	SCN5A
MOL000358	beta-sitosterol	Gamma-aminobutyric-acid receptor alpha-2 subunit	
MOL000358	beta-sitosterol	Muscarinic acetylcholine receptor M4	CHRM4
MOL000358	beta-sitosterol	CGMP-inhibited 3',5'-cyclic phosphodiesterase A	PDE3A
MOL000358	beta-sitosterol	5-hydroxytryptamine 2A receptor	HTR2A
MOL000358	beta-sitosterol	Gamma-aminobutyric-acid receptor alpha-5 subunit	
MOL000358	beta-sitosterol	Alpha-1A adrenergic receptor	ADRA1A
MOL000358	beta-sitosterol	Gamma-aminobutyric-acid receptor alpha-3 subunit	

MOL000358	beta-sitosterol	Muscarinic acetylcholine receptor M2	CHRM2
MOL000358	beta-sitosterol	Alpha-1B adrenergic receptor	ADRA1B
MOL000358	beta-sitosterol	Beta-2 adrenergic receptor	ADRB2
MOL000358	beta-sitosterol	Neuronal acetylcholine receptor subunit alpha-2	CHRNA2
MOL000358	beta-sitosterol	Sodium-dependent serotonin transporter	SLC6A4
MOL000358	beta-sitosterol	Mu-type opioid receptor	OPRM1
MOL000358	beta-sitosterol	Gamma-aminobutyric acid receptor subunit alpha-1	GABRA1
MOL000358	beta-sitosterol	Neuronal acetylcholine receptor protein, alpha-7 chain	
MOL000358	beta-sitosterol	Cytochrome P450-cam	
MOL000358	beta-sitosterol	Apoptosis regulator Bcl-2	BCL2
MOL000358	beta-sitosterol	Apoptosis regulator BAX	BAX
MOL000358	beta-sitosterol	Caspase-9	CASP9
MOL000358	beta-sitosterol	Transcription factor AP-1	JUN
MOL000358	beta-sitosterol	Caspase-3	CASP3
MOL000358	beta-sitosterol	Caspase-8	CASP8
MOL000358	beta-sitosterol	Protein kinase C alpha type	PRKCA
MOL000358	beta-sitosterol	Transforming growth factor beta-1	TGFB1
MOL000358	beta-sitosterol	Serum paraoxonase/arylesterase 1	PON1
MOL000358	beta-sitosterol	Microtubule-associated protein 2	MAP2
MOL000359	sitosterol	Progesterone receptor	PGR
MOL000359	sitosterol	Nuclear receptor coactivator 2	NCOA2
MOL000359	sitosterol	Mineralocorticoid receptor	NR3C2
MOL004355	Spinasterol	Progesterone receptor	PGR
MOL004355	Spinasterol	Mineralocorticoid receptor	NR3C2
MOL004355	Spinasterol	Nuclear receptor coactivator 2	NCOA2
MOL000449	Stigmasterol	Progesterone receptor	PGR
MOL000449	Stigmasterol	Mineralocorticoid receptor	NR3C2
MOL000449	Stigmasterol	Nuclear receptor coactivator 2	NCOA2
MOL000449	Stigmasterol	Alcohol dehydrogenase 1C	ADH1C
MOL000449	Stigmasterol	Ig gamma-1 chain C region	
MOL000449	Stigmasterol	Retinoic acid receptor RXR-alpha	RXRA
MOL000449	Stigmasterol	Nuclear receptor coactivator 1	NCOA1
MOL000449	Stigmasterol	Prostaglandin G/H synthase 1	PTGS1
MOL000449	Stigmasterol	Prostaglandin G/H synthase 2	PTGS2
MOL000449	Stigmasterol	Alpha-2A adrenergic receptor	ADRA2A
MOL000449	Stigmasterol	Sodium-dependent noradrenaline	SLC6A2

		transporter	
MOL000449	Stigmasterol	Sodium-dependent dopamine transporter	SLC6A3
MOL000449	Stigmasterol	Beta-2 adrenergic receptor	ADRB2
MOL000449	Stigmasterol	Aldose reductase	AKR1B1
MOL000449	Stigmasterol	Urokinase-type plasminogen activator	PLAU
MOL000449	Stigmasterol	Leukotriene A-4 hydrolase	LTA4H
MOL000449	Stigmasterol	Amine oxidase [flavin-containing] B	MAOB
MOL000449	Stigmasterol	Amine oxidase [flavin-containing] A	MAOA
MOL000449	Stigmasterol	mRNA of PKA Catalytic Subunit C-alpha	
MOL000449	Stigmasterol	Chymotrypsinogen B	CTRB1
MOL000449	Stigmasterol	Muscarinic acetylcholine receptor M3	CHRM3
MOL000449	Stigmasterol	Muscarinic acetylcholine receptor M1	CHRM1
MOL000449	Stigmasterol	Beta-1 adrenergic receptor	ADRB1
MOL000449	Stigmasterol	Sodium channel protein type 5 subunit alpha	SCN5A
MOL000449	Stigmasterol	5-hydroxytryptamine 2A receptor	HTR2A
MOL000449	Stigmasterol	Alpha-1A adrenergic receptor	ADRA1A
MOL000449	Stigmasterol	Gamma-aminobutyric-acid receptor alpha-3 subunit	
MOL000449	Stigmasterol	Muscarinic acetylcholine receptor M2	CHRM2
MOL000449	Stigmasterol	Alpha-1B adrenergic receptor	ADRA1B
MOL000449	Stigmasterol	Gamma-aminobutyric acid receptor subunit alpha-1	GABRA1
MOL000449	Stigmasterol	Neuronal acetylcholine receptor protein, alpha-7 chain	
MOL000492	(+)-catechin	Prostaglandin G/H synthase 1	PTGS1
MOL000492	(+)-catechin	Estrogen receptor	ESR1
MOL000492	(+)-catechin	Estrogen receptor	ESR1
MOL000492	(+)-catechin	Prostaglandin G/H synthase 2	PTGS2
MOL000492	(+)-catechin	Heat shock protein HSP 90	HSP90AA1
MOL000492	(+)-catechin	Heat shock protein HSP 90	HSP90AA1
MOL000492	(+)-catechin	Beta-lactamase	
MOL000492	(+)-catechin	mRNA of PKA Catalytic Subunit C-alpha	
MOL000492	(+)-catechin	Nuclear receptor coactivator 2	NCOA2

MOL000492	(+)-catechin	Calmodulin	CALM1
MOL000492	(+)-catechin	Calmodulin	CALM3
MOL000492	(+)-catechin	Retinoic acid receptor RXR-alpha	RXRA
MOL000492	(+)-catechin	Catalase	CAT
MOL000492	(+)-catechin	Hyaluronan synthase 2	HAS2
MOL006990	(1S,2S,4R)-trans-2-hydroxy-1,8-cineole-B-D-glucopyranoside	N/A	
MOL006992	(2R,3R)-4-methoxyl-distylin	Nitric oxide synthase, inducible	NOS2
MOL006992	(2R,3R)-4-methoxyl-distylin	Prostaglandin G/H synthase 1	PTGS1
MOL006992	(2R,3R)-4-methoxyl-distylin	Estrogen receptor	ESR1
MOL006992	(2R,3R)-4-methoxyl-distylin	Estrogen receptor	ESR1
MOL006992	(2R,3R)-4-methoxyl-distylin	Prostaglandin G/H synthase 2	PTGS2
MOL006992	(2R,3R)-4-methoxyl-distylin	Heat shock protein HSP 90	HSP90AA1
MOL006992	(2R,3R)-4-methoxyl-distylin	Heat shock protein HSP 90	HSP90AB1
MOL006994	1-o-beta-d-glucopyranosyl-8-o-benzoylpaeonisuffrone_qt	N/A	
MOL006996	1-o-beta-d-glucopyranosylpaeonisuffrone_qt	N/A	
MOL006999	stigmast-7-en-3-ol	Progesterone receptor	PGR
MOL007003	benzoyl paeoniflorin	N/A	
MOL007004	Albiflorin	N/A	
MOL007005	Albiflorin_qt	N/A	
MOL007008	4-ethyl-paeoniflorin_qt	N/A	
MOL007012	4-o-methyl-paeoniflorin_qt	N/A	
MOL007014	8-debenzoylpaeonidanin	N/A	
MOL007016	Paeoniflorigenone	N/A	
MOL007018	9-ethyl-neo-paeoniaflorin A_qt	N/A	
MOL007022	evofolinB	N/A	
MOL007025	isobenzoylpaeoniflorin	N/A	
MOL002883	Ethyl oleate (NF)	Nuclear receptor coactivator 2	NCOA2
MOL005043	campest-5-en-3beta-ol	Progesterone receptor	PGR
