# Supplementary material for: Analysis of the Mechanism of GuizhiFuling Wan in Treating Adenomyosis Based on Network Pharmacology Combined with Molecular Docking and Experimental Verification
Source: Evid Based Complement Alternat Med. 2022 Aug 26;2022:6350257. doi: 10.1155/2022/6350257 (PMC9440632; doi:10.1155/2022/6350257)
Supplement: Supplementary Materials — Figure S1: HPLC figure of baicalein, β-sitosterol, and stigmasterol. Table S1: GFW-related compounds and targets. Table S2: AM-related targets. Table S3: GFW-AM common targets. Table S4: GFW-AM common targets' string interactions and key targets. [file 6350257.f1.zip › Supplementary Table S2.pdf]

Supplementary Table S2 AM related targets

MMP2
VEGFA
ESR1
MMP9
CYP19A1
PGR
PTGS2
OXTR
CTNNB1
TIMP2
SPP1
NGF
COMT
GPER1
TP53
HOXA10
CXCL8
PRL
CDH1
MME
IL6
ESR2
LIF
GNRH1
OXT
MIR10B
APC
MAPK8IP1
CCR6
HNF1B
SERPINA3
U2AF1
MAPK8IP2
H2AC18
MIR21
MIR146A
MIR140
MIR126
MIR34A
MIR27A
MIR143
MIR145

MIR149
MIR141
MIR124-1
MIR125A
MIR106B
MIR10A
MIR34C
MIR200A
MIR204
MIR210
MIR29A
MIR30C1
MIR30E
MIR99A
MIRLET7A1
MIRLET7D
MIR17
MIR182
MIR193B
MIR196B
MIR200B
MIR205
MIR214
MIR23B
MIR25
MIR30B
MIR31
MIR22
MIR221
MIR127
MIR142
MIR30A
MIR9-1
MIR93
MIRLET7I
MIR181C
MIR146B
MIR139
MIR195
MIR196A1
MIR181B1
MIR155
MIR15B
MIR101-1

MIR183
MIR186
MIR193A
MIR423
MIR29C
MIR324
MIR330
MIR331
MIR199A1
MIR520A
MIR590
MIR335
MIR483
MIR455
MIR218-1
MIR224
MIR675
MIR338
MIR103A1
MIR542
MIR15A
MIR371A
MIR361
MIR144
MIR486-1
MIR512-1
MIR20B
MIR517A
MIR744
MIR509-1
MIR518D
ANXA2
MUC16
BCL2
MMP1
MMP3
MSN
ROCK1
PAK4
MSI1
NTRK1
NGFR
ITGB3
IL10

STIP1
PAK1
NOS3
CYP1A1
RHOA
CAV1
CCR1
KISS1
NCAM1
NTF3
DUSP6
SPRY4
NDUFA13
IL17RD
EGF
PTEN
DNMT3B
PPP2CA
BIRC5
CNR1
NCOA3
CNR2
NCOA2
PDCD4
EIF3E
IL37
MDM2
DNMT3A
KRAS
LIFR
NR4A1
ARHGAP26
IGFBP1
CCN1
EGFR
NOTCH1
BAX
ALOX5
NUMB
YWHAQ
SNAI1
MTOR
TLR4
NFE2L2

ILK
CYP1A2
PARK7
FMNL2
TIMP1
MMP7
ACVR2B
FST
ACVR2A
MSTN
HMOX1
HMOX2
CXCL1
IL22
CDKN2A
CCL2
CXCR2
CXCR1
PPARG
MEGF10
NTRK2
FGF1
FGF2
IL18
STS
IL18R1
IL18BP
SOD2
CYP17A1
HIF1A
BECN1
AVPR1A
CSF2
KIR3DL1
EBAG9
KIR2DL3
ITGA6
MLH1
ITGA4
ITGA5
XDH
HLA-DRB1
TRPV1
ITGA2

GH1
HLA-DQA1
CAT
GJA1
CDKN1A
HLA-G
ELN
PNOC
NOS2
AKT1
PIK3CA
NFKB1
NFKBIA
RXRA
IGF2
INHA
ACTC1
INTS9
PCNA
PTGIS
PTGER3
TIMP3
LAMC2
ADM
MKI67
CALB1
GSTM1
TIMP4
LINC-ROR
NINJ1
EZH2
TGFB1
EGR1
CCN2
TUG1
ROCK2
CCND1
RB1
ZEB1
LCN2
IFITM1
BGLAP
SLC2A1
PLAU

IFNG
ENG
PLAUR
S100A13
CDH2
NR5A1
VIM
PLAT
DES
IL1B
GNRHR
BMP2
IRS1
CLU
IL4
BMP7
SLC2A4
TNFSF13B
FHIT
MED12
SERPINB2
HSD17B2
IL5
BMP6
PTTG1
HOXA11
SIK3
KRT7
MLANA
RBP1
ADAMTS9
FNDC5
TNF
TBX1
NBR1
F3
HGF
GSTM2
GABPA
