# Supplementary material for: Analysis of the Mechanism of GuizhiFuling Wan in Treating Adenomyosis Based on Network Pharmacology Combined with Molecular Docking and Experimental Verification
Source: Evid Based Complement Alternat Med. 2022 Aug 26;2022:6350257. doi: 10.1155/2022/6350257 (PMC9440632; doi:10.1155/2022/6350257)
Supplement: Supplementary Materials — Figure S1: HPLC figure of baicalein, β-sitosterol, and stigmasterol. Table S1: GFW-related compounds and targets. Table S2: AM-related targets. Table S3: GFW-AM common targets. Table S4: GFW-AM common targets' string interactions and key targets. [file 6350257.f1.zip › Supplementary Table S3.pdf]

Supplementary Table S3 GFW-AM common targets

AKT1
TP53
IL6
TNF
VEGFA
MMP9
PTGS2
ESR1
HIF1A
MMP2
TGFB1
CAT
CXCL8
PPARG
NOS2
CDKN1A
PGR
PLAU
NFKBIA
IGF2
NCOA2
BAX
RXRA
BCL2
GSTM1
GSTM2
