# Supplementary material for: Analysis of the Mechanism of GuizhiFuling Wan in Treating Adenomyosis Based on Network Pharmacology Combined with Molecular Docking and Experimental Verification
Source: Evid Based Complement Alternat Med. 2022 Aug 26;2022:6350257. doi: 10.1155/2022/6350257 (PMC9440632; doi:10.1155/2022/6350257)
Supplement: Supplementary Materials — Figure S1: HPLC figure of baicalein, β-sitosterol, and stigmasterol. Table S1: GFW-related compounds and targets. Table S2: AM-related targets. Table S3: GFW-AM common targets. Table S4: GFW-AM common targets' string interactions and key targets. [file 6350257.f1.zip › Supplementary Table S4.pdf]

Supplementary Table S4 GFW-AM common targets string interactions and key targets

name	Degree	BetweennessCentrality	ClosenessCentrality
AKT1	23	0.06340195	0.92592593
TP53	23	0.09763163	0.92592593
IL6	20	0.02108954	0.83333333
TNF	20	0.03354312	0.83333333
VEGFA	20	0.02251811	0.83333333
MMP9	19	0.01356573	0.80645161
PTGS2	18	0.01031405	0.78125
ESR1	18	0.02372959	0.78125
HIF1A	18	0.02179914	0.78125
MMP2	17	0.00727448	0.75757576
TGFB1	17	0.00652954	0.75757576
CAT	17	0.10347767	0.75757576
CXCL8	17	0.00675681	0.75757576
PPARG	16	0.01616835	0.73529412
NOS2	14	0.00866306	0.69444444
CDKN1A	14	0.00636147	0.67567568
PGR	12	0.00489971	0.64102564
PLAU	12	9.39E-04	0.64102564
NFKBIA	11	0.00400144	0.625
IGF2	11	9.09E-04	0.625
NCOA2	8	0.00536644	0.58139535
BAX	7	0.00360562	0.58139535
RXRA	7	0.00569841	0.55555556
BCL2	6	0.0042316	0.55555556
GSTM1	3	0.00419072	0.52083333
GSTM2	2	0	0.44642857

GFW-AM key targets

AKT1
TP53
IL6
TNF
VEGFA
MMP9
PTGS2
ESR1
HIF1A
MMP2
TGFB1
CAT
CXCL8
