# Supplementary figures and images for: Analysis of the Mechanism of GuizhiFuling Wan in Treating Adenomyosis Based on Network Pharmacology Combined with Molecular Docking and Experimental Verification
Source: Evid Based Complement Alternat Med. 2022 Aug 26;2022:6350257. doi: 10.1155/2022/6350257 (PMC9440632; doi:10.1155/2022/6350257)

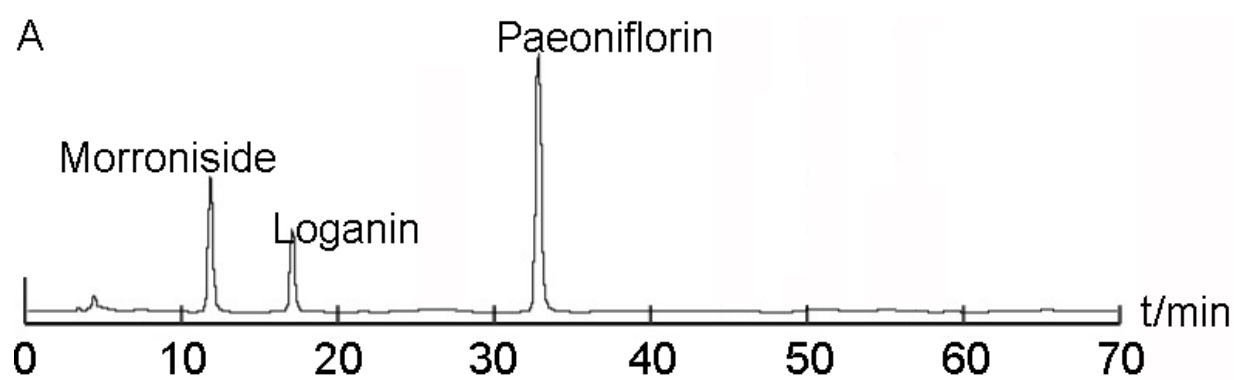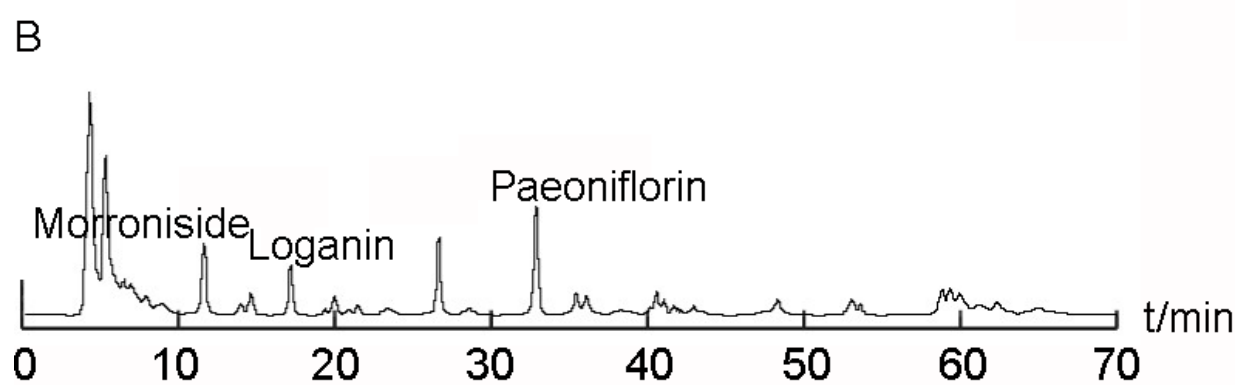

Figure S1 The results of HPLC (A: standard solution; B: MTHSWD solution)

Supplement: Supplementary Materials — Figure S1: HPLC figure of baicalein, β-sitosterol, and stigmasterol. Table S1: GFW-related compounds and targets. Table S2: AM-related targets. Table S3: GFW-AM common targets. Table S4: GFW-AM common targets' string interactions and key targets. [file 6350257.f1.zip › Figure S1.pdf]
